# Life stage and tissue speciation of cathepsin B (AGAP004533) derives different functional properties in the G3 strain of the mosquito *Anopheles gambiae*

**DOI:** 10.1186/1753-6561-6-S6-P1

**Published:** 2012-10-01

**Authors:** Daniel Achinko, Dan Masiga, Paul Mireji, Flaminia Catteruccia

**Affiliations:** 1 Molecular Biology and Biotechnology Department, International Center of Insect Physiology and Ecology, Nairobi, Kenya, PO Box 30772-00100; 2 Biochemistry Department, Egerton University, Njoro, Kenya, PO Box 20115-536; 3 Dipartimento di Medicina Sperimentale e Scienze Biochimiche, Università degli Studi di Perugia, 05100 Terni, Italy

## 

Cathepsin B is a lysosomal papain-like cysteine peptidase that is expressed in all tissues and functions primarily as an exopeptidase through its carboxydipeptidyl activity. Together with other cathepsins, it is involved in the degradation of proteins, proenzyme activation, antigen processing, metabolism and apoptosis. AGAP004533 is a cathepsin B peptidase of 337 amino acids known to be found on the mating plug. This plug is known to be produced in the male *Anopheles gambiae* mosquito and transferred to the female during mating [1]. The female digests this plug in 24 h. The protein is expressed in all life stages of the mosquito and in all tissues of the adult. We cloned and sequenced the protein in the larvae and pupae stages and all reproductive tissues (spermatheca, atria and ovary of the female; testes and male accessory glands (MAGs) of the male) of the G3 mosquito strain. These sequences were analysed with Geneious 5.5.5 and cLc workbench 6.6.1 software. Within the coding sequence, two single mutations at C584T (juvenile stages) and nucleotide A14T (ovary) were identified. The latter translates into a glutamine for leucine (Q6L), which causes the loss of the signal peptide due to loss of five amino acids at the N-terminal region of the protein sequence, meanwhile the former translates into an alanine for valine (A195V). Both mutations cause structural modifications within the secondary structure of the protein that eventually affect its 3D conformation. The sequences in the artria and spermatheca showed insertion of a cytosine at nucleotide 1010, which translates to a proline for a leucine (P337L) substitution, and hence loss of the stop codon at amino acid 338. This loss causes an extension of 14 amino acids at the carboxylic end of the protein, resulting in secondary structure modification. The sequence for the testes appeared transposed, and hence was not considered in the analysis. All the sequences translated on the same frame except for that of the ovary, which translated on a different frame. Protein BLAST of these sequences at NCBI using the blosum62 matrix, identified with AGAP004533 of *Anopheles gambiae* alongside other mosquito species, although that of the artria and spermatheca also identified with species of distant taxa such as *Manduca sexta* (FM957999.1) and *Gallus gallus* (NM205371.1). This relation was due to the amino acid extensions at the carboxylic end relating to parasite killing in the former, and embryonic apoptosis in the latter. Transcription factor predictions on all sequences identified equal binding sites (T00821, T00752), and that of the male accessory glands identified an extra binding site (T00360) known in humans as a bifunctional protein nuclear cytoplasmic *O*-*N*-acetylglucosaminidase and acetyltransferase. This site also has alternative splicing functions, which could be important for the variations observed in this gene. Sequence variations of this protein in the different stages and tissues of the mosquitoes may also be highly related to their functions and relative positions in the same or different biological processes within the various tissues. In-depth analysis of the reproductive role of AGAP004533 will help in reproductive control of the vector.
